# Revealing Clonal Responses of Tumor-Reactive T-Cells Through T Cell Receptor Repertoire Analysis

**DOI:** 10.3389/fimmu.2022.807696

**Published:** 2022-01-27

**Authors:** Hiroyasu Aoki, Shigeyuki Shichino, Kouji Matsushima, Satoshi Ueha

**Affiliations:** ^1^ Division of Molecular Regulation of Inflammatory and Immune Diseases, Research Institute for Biomedical Sciences, Tokyo University of Science, Chiba, Japan; ^2^ Department of Hygiene, Graduate School of Medicine, The University of Tokyo, Tokyo, Japan

**Keywords:** CD8^+^ T cell, T-cell receptor repertoire, single-cell TCR-seq, immune check inhibitors, cancer-immunity cycle, inter-organ clone tracking

## Abstract

CD8^+^ T cells are the key effector cells that contribute to the antitumor immune response. They comprise various T-cell clones with diverse antigen-specific T-cell receptors (TCRs). Thus, elucidating the overall antitumor responses of diverse T-cell clones is an emerging challenge in tumor immunology. With the recent advancement in next-generation DNA sequencers, comprehensive analysis of the collection of TCR genes (TCR repertoire analysis) is feasible and has been used to investigate the clonal responses of antitumor T cells. However, the immunopathological significance of TCR repertoire indices is still undefined. In this review, we introduce two approaches that facilitate an immunological interpretation of the TCR repertoire data: inter-organ clone tracking analysis and single-cell TCR sequencing. These approaches for TCR repertoire analysis will provide a more accurate understanding of the response of tumor-specific T cells in the tumor microenvironment.

## Introduction

Each T cell has a unique antigen receptor (T-cell receptor; TCR). TCR is composed of alpha and beta chains, whose specificity is determined by the gene rearrangement that occurs in the thymus. T cells specifically recognize their cognate antigens *via* the TCRs and are activated. These activated T cells proliferate in the periphery, eliciting responses against the corresponding antigen, including cancer antigens. Conventional preclinical and clinical studies on antitumor T-cell responses have analyzed a limited number of tumor-specific T-cell clones using TCR transgenic mice (1) or peptide-major histocompatibility complex multimer technology (2). However, it is becoming increasingly clear that antitumor T-cell responses are driven by a wide variety of T-cell clones (3, 4).

The collection of TCRs *in vivo*, termed as the TCR repertoire, is considered to be a new indicator to evaluate T-cell responses based on antigen specificity. Recent advances in next-generation sequencing (NGS) technology have enabled TCR sequencing (TCRseq), which allows a comprehensive determination of the TCR sequences in individuals (5). TCR repertoire analysis is currently being used for studies in various medical fields, including infectious diseases (6), transplantation immunity (7), and tumor immunity (8). In this review, we provide an overview of the TCRseq methods, and summarize the findings and limitations of current TCR repertoire analysis in the field of cancer immunotherapy. Additionally, we introduce two novel approaches in TCR repertoire analysis that facilitate the immunological interpretation of TCR repertoire data: inter-organ clone tracking analysis, which identifies and analyzes T-cell clones present in the tumor and other tissues, and single-cell (sc) TCRseq, which identifies TCR sequences and their gene expression profiles at a single cell resolution.

## Overview of TCR Sequencing Methods

**Figure 1:**
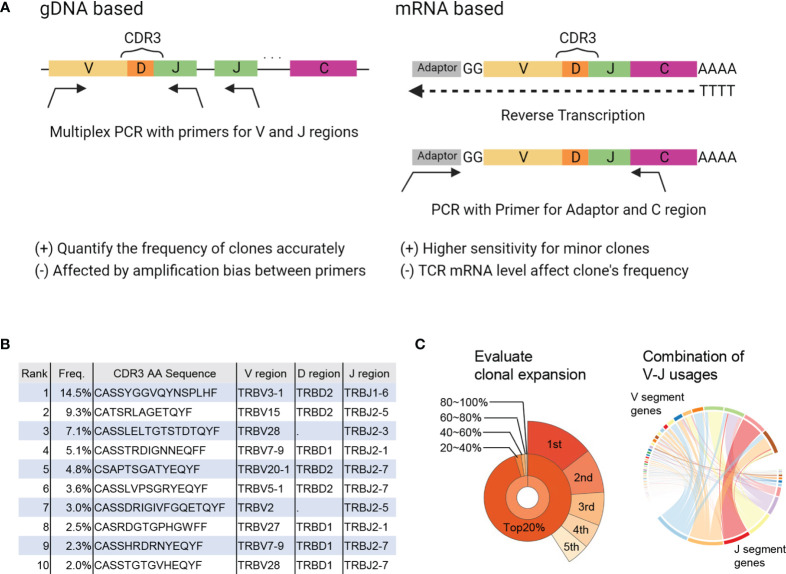
Summary of TCRseq and TCR repertoire analysis. **(A)** Exemplary workflow of TCR library preparation methods. Genomic (g) DNA-based methods use a primer set designed to cover all V or J regions. Multiplex PCR is used to amplify CDR3 generated by V(D)J recombination. In the messenger (m)RNA-based method, an adaptor sequence is attached to the 5′ end of mRNA in the reverse transcription reaction. Then, TCR cDNA containing CDR3 is amplified using primers targeting the C region and adaptor sequence. **(B)** Output of primary analysis: table of T-cell clones with their frequency, CDR3 sequence, and usage of V, D, and J gene segments. **(C)** Output of secondary analysis. (left) Pie chart of the frequencies of top1–5 clones (outer layer) and total frequencies of top20%, 20–40%, 40–60%, 60–80%, and <80% of clones (inner layer). (right) Circos plot representing the proportion of each combination of V-J segment usage. The widths of the ribbons connecting V (upper side) and J (lower side) segments correspond to the total frequency of clones with the given combination of V-J segment usage.

TCRs are produced through rearrangement of the variable (V), diversity (D), joining (J), and constant (C) gene segments as well as insertions and deletions, resulting in a vast diversity (9). In TCRseq, the TCR gene, including complementary determining region 3 (CDR3), which is the most variable region in the TCR and a significant contributor to antigen specificity, is amplified and then sequenced by NGS. There are several TCRseq methods, which can be classified based on (i) whether genomic DNA (gDNA) or messenger RNA (mRNA) is used as a template, and (ii) whether multiple V region-specific primers are used to amplify the TCR sequence (multiplex PCR) or universal primers for the adapter sequence are used (5′ rapid amplification of cDNA ends [RACE]) (10) (**Figure 1A**). Because each method has its advantages and disadvantages (**Table 1**) (11, 12), it is preferable to adopt the most appropriate TCRseq method for a given research purpose based on these characteristics. In addition, the impact of each TCRseq method on the repertoire data should be considered when comparing TCR repertoire data obtained by different methods.

**Table 1:** Classifications of TCRseq methods and their characteristics.

Starting materials	Amplification of TCR sequences	Advantage, disadvantage	Ref.
gDNA-based	Multiplex PCR	(+) Accurate quantification of clonal composition	(11)
		(-) Lower detection sensitivity due to small number of templates per cell	
mRNA-based	Multiplex PCR	(+) higher detection sensitivity	(12)
		(+) No need for appending adapter sequences for amplification	
mRNA-based	5′ RACE	(+) Not affected by amplification bias between V regions	(12)

TCR sequences obtained by NGS are aligned to reference sequences of the V, D, and J regions and then assembled into T-cell clones that share the same CDR3 sequence. Algorithms, such as IgBLAST (13), IMGT/HighV-QUEST (14), MiXCR (15), and RTCR (16) are used to analyze TCR sequence data (**Table 2**). The results are presented in a table summarizing all detected T-cell clones and their frequencies (primary analysis, **Figure 1B**). Based on this clone table, the characteristics of TCR repertoires, including diversity (i.e., the number of T-cell clones present in the repertoire) and clonality (i.e., the extent to which specific T-cell clones are expanded in the repertoire), can be evaluated (secondary analysis, **Figure 1C**).

**Table 2:** Computational tools for TCR-seq data analysis.

Tools	Ref.	Citations^A^	Usage	Link for documentation web page
IgBLAST	(13)	722	Bulk TCRseq	https://ncbi.github.io/igblast/
IMGT/HighV-QUEST	(14)	360	Bulk TCRseq	http://www.imgt.org/HighV-QUEST/doc.action;jsessionid=4F133D1C9F164A0E88D5F99FE16D118A
MiXCR	(15)	815	Bulk TCRseq	https://mixcr.readthedocs.io/en/master/
RTCR	(16)	39	Bulk TCRseq	https://github.com/uubram/RTCR
TraCeR	(17)	328	TCR reconstruction from scRNAseq	https://github.com/Teichlab/tracer
VDJPuzzle	(18)	57	TCR reconstruction from scRNAseq	https://github.com/simone-rizzetto/VDJPuzzle

^A^Citation statistics were obtained from Google Scholar before December 21, 2021 (https://scholar.google.com/).

## TCR Repertoire Analysis in Tumor Immunology: Achievements and Challenges

As cancer immunotherapies, such as anti-CTLA4 antibody and PD-1 inhibitor therapies, are applied to a wide range of cancer types, biomarkers that can predict treatment responders are urgently required (19). Treatment responses are reportedly associated with the level of PD-L1 expression in the tumor (20) and the mismatch repair deficiency (MMRd) of the tumor (21, 22), which are tumor-related factors. However, predictive biomarkers reflecting the patient’s immune status have not been established to date. TCR repertoire has been suggested as a candidate. The association between TCR repertoires and therapeutic effects in patients receiving cancer immunotherapy was reported around 2014, as summarized in **Table 3**. A clinical study on anti-PD-1 antibody therapy in melanoma patients showed that the clonality of the TCR repertoire in the tumor before treatment was higher in responders than in non-responders (23–26). Similarly, the total frequency of the top 1% clones in tumor repertoire was greater in responders than in non-responders in studies on neoadjuvant immunotherapy in patients with non-small cell lung cancer (NSCLC) (27, 28). Furthermore, responders showed more T-cell clones with increased frequency (23) and increased clonality of the T-cell repertoire (29) in the tumor after treatment. These results suggest that the clonality of the TCR repertoire in the tumor may reflect and predict the therapeutic effect of cancer immunotherapy. However, CD4^+^ and CD8^+^ tumor-infiltrating lymphocytes (TILs) were not separated prior to TCRseq in these studies. Considering that CD8^+^ TILs generally exhibit higher clonality than CD4^+^ (35, 36), the higher clonality of the total TIL repertoire may simply reflect a higher proportion of CD8^+^ cells (37).

**Table 3:** Previous studies analyzing the association between repertoire clonality/diversity and clinical responses.

Authors	Ref.	Year	Tumor^A^	Treat	Organ	Repertoire indices associated with better responses
Tumeh et al.	(23)	2014	Melanoma	aPD1	Tumor	Higher clonality at pre-treatment
						More clones with increased frequency during treatment.
Roh et al.	(24)	2017	Melanoma	aPD1 aCTLA4	Tumor	Higher clonality at pre- and post-treatment (aPD1).
Yusko et al.	(25)	2019	Melanoma	aPD1 aCTLA4	Tumor	Higher clonality at pre-treatment (aPD1).
Valpione et al.	(26)	2021	Melanoma	aPD1	Tumor	Higher clonality at pre-treatment.
Zhang et al.	(27)	2019	NSCLC	Neoadjuvant aPD1	Tumor	Higher clonality at post-treatment.
Casarrubios et al.	(28)	2021	NSCLC	Neoadjuvant aPD1+chemo	Tumor	Higher total frequency of top 1% clones at pre-treatment.
Riaz et al.	(29)	2017	Melanoma	aPD1 aCTLA4	Tumor	Increase in clonality during treatment (aPD1).
Cha et al.	(30)	2014	Prostate Melanoma	aCTLA4	Blood	Less clones with decreased frequency during treatment.
Fiarfax et al.	(31)	2020	Melanoma	aPD1 aCTLA4	Blood	More large clones at post-treatment.
Kato et al.	(32)	2021	RCC	aPD1	Blood	Decreased diversity at post-treatment.
Dong et al.	(33)	2021	NSCLC	aPD1 Chemo	Blood	Increased diversity during treatment.
Naidus et al.	(34)	2020	NSCLC	aPDL1	Blood	Decreased clonality during treatment.

^A^NSCLC, Non-small cell lung cancer; RCC, Renal cell carcinoma.

As blood sampling is less invasive than a tumor biopsy, peripheral blood samples are suitable for long-term immune monitoring of patients with cancer. Cha et al. reported that responders among prostate cancer and melanoma patients treated with anti-CTLA4 antibody had fewer contracted T-cell clones in the peripheral blood after treatment (30). Fairfax et al. found that responders among melanoma patients treated with anti-PD-1 antibody alone or in combination with anti-CTLA4 antibody had higher numbers of expanded CD8^+^ T-cell clones in their peripheral blood (31). A similar trend has been reported in a cohort of renal cell carcinoma patients treated with anti-PD-1 antibodies (32). In contrast, responders among NSCLC patients showed decreased clonality and increased diversity in peripheral blood TCR repertoires after treatment (33, 34). Therefore, there is currently no consensus on the characteristics of peripheral blood TCR repertoires associated with therapeutic responses. This is because the diversity and clonality of the TCR repertoire vary greatly among individuals and the peripheral blood comprises a large fraction of T-cell clones that are not related to the antitumor immune response.

Despite these issues, the diversity and clonality of the TCR repertoire are becoming recognized as factors associated with prognosis and/or therapeutic effects. However, the immunopathological significance of these TCR repertoire indices is still undefined because clonality or diversity as determined by TCR repertoire analysis does not consider the immunological features of individual T-cell clones. In other words, whether a clone is CD8^+^ or CD4^+^, tumor-reactive or bystander, effector or exhausted, is ignored, and only the frequency distribution of clones is analyzed. Therefore, we introduce two approaches in TCR repertoire analysis to support the immunological interpretation of TCR repertoire data. The first approach is inter-organ clone tracking analysis, in which T-cell clones in the tumor are tracked in the blood and/or draining lymph node (dLN). The second is single-cell (sc) TCRseq, in which gene expression profile is integrated into individual T-cell clones.

## TCR Repertoire Analysis Based on Inter-Organ Overlapping Clones

**Figure 2:**
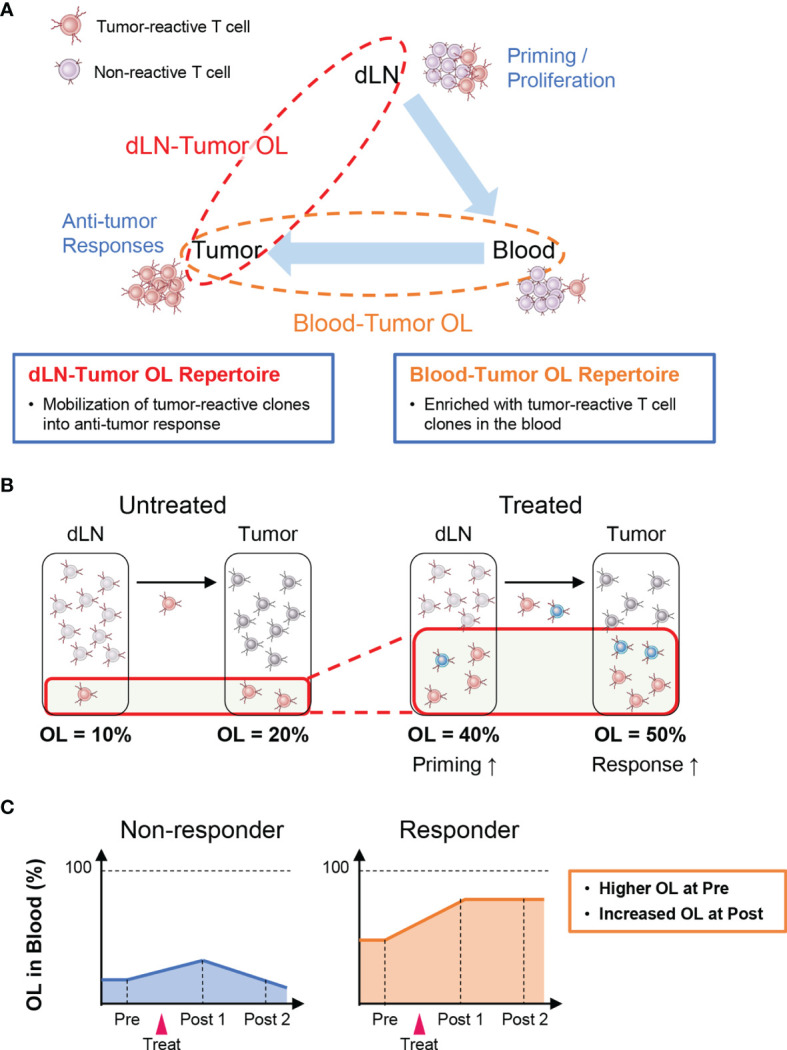
Outline of inter-organ clone tracking analysis. **(A)** Tumor-reactive T cells are activated and proliferate after antigen presentation in tumor-draining lymph nodes (dLNs) and then infiltrate into the tumor tissue *via* the blood circulation. Therefore, the dLN-tumor overlapping (OL) repertoire reflects a novel mobilization of tumor-reactive clones into the antitumor T-cell response. In addition, the blood-tumor OL repertoire is expected to be enriched in tumor-reactive T-cell clones. **(B)** Overview of dLN-tumor overlapping repertoire analysis. An increase in overlapping clones is associated with enhanced priming of tumor-reactive clones and stronger antitumor effects. **(C)** Overview of blood-tumor OL repertoire analysis. Patients with a higher blood-tumor overlap in the blood at baseline and an increase in blood-tumor overlap following treatment show a better antitumor response.

Tumor-reactive T cells are activated and proliferate after antigen presentation in lymph nodes where cancer antigens enter (i.e., dLNs) and then infiltrate into the tumor tissue *via* the peripheral blood (Cancer Immunity Cycle: 38). Therefore, T-cell clones detected in both the tumor and the dLNs (dLN-tumor overlapping repertoire) represent tumor-reactive clones that are mobilized for the antitumor T-cell response (**Figure 2A**). Thus, an increase in overlapping clones is considered to be associated with enhanced priming of tumor-reactive clones and stronger antitumor effects (**Figure 2B**). In the peripheral blood, there exist many T-cell clones that are unrelated to the antitumor response. Hence, the TCR repertoire in the peripheral blood that overlaps with that in the tumor (peripheral blood-tumor overlapping repertoire) is expected to be enriched in tumor-reactive T-cell clones. Therefore, the blood-tumor overlapping repertoire may be useful for the monitoring of antitumor T-cell responses. For example, patients with a higher blood-tumor overlap in the blood at baseline and an increase in the blood-tumor overlap following treatment may exhibit a better antitumor response (**Figure 2C**). Moreover, blood-tumor overlapping clones in the unfractionated tumor T-cell repertoire can be annotated as CD4^+^ or CD8^+^ based on whether overlapping clones are detected in the blood CD4^+^ or CD8^+^ T-cell repertoire. Given that isolating sufficient numbers of CD4^+^ and CD8^+^ T cells from tumor biopsy is technically difficult and laborious, the ability to analyze the responses of CD4^+^ and CD8^+^ T-cell clones in the tumor without the need for preparing CD4^+^ and CD8^+^ TILs is another advantage of blood-tumor overlapping repertoire analysis.

### dLN-Tumor Overlapping Repertoire Analysis

A few studies have performed dLN-tumor overlapping analysis on samples surgically resected from patients with cancer (**Table 4**). Inamori et al. reported that patients with MMRd colorectal cancer (CRC), for which ICIs are more effective, exhibited a greater extent of dLN-tumor overlap than those with MMR proficient CRC (39). This result is consistent with that of MMRd tumors harboring tumor-reactive T-cell clones specific to mutation-associated neoantigens (22). Considering this result, other tumor types that exhibit greater dLN-tumor overlap may also be responsive to ICIs. However, it is noted that metastasis-positive dLN showed a significantly higher extent of dLN-tumor overlap (40, 41). Thus, the degree of overlap between metastasis-positive dLN and the tumor may not necessarily reflect the enhanced priming of tumor-reactive clones.

**Table 4:** Previous studies performing bulk TCR sequencing inter-organ clone tracking analysis.

Authors	Ref.	Year	Tumor^A^	Treat	Organ^B^	Findings
Inamori et al.	(39)	2021	CRC	(-)	dLN	MMRd CRC showed a higher extent of dLN-tumor overlap.
Wang et al.	(40)	2016	Breast	(-)	dLN	Freq. of dLN-tumor overlapping clones was higher in tumor.
Matsuda et al.	(41)	2019	CRC	(-)	dLN	Metastasis-positive dLNs have a greater extent of dLN-tumor overlap.
Aoki et al.	(42)	2019	B16 (mouse)	aCD4	dLN	dLN-tumor overlapping repertoire was increased by anti-CD4.
Chow et al.	(43)	2020	RCC	SBRT	Blood	Tumor-enriched clones expanded in blood within 2 weeks post-treatment.
Aoki et al.	(44)	2021	GI	aPD1	Blood	Tumor-overlapping clones in blood were higher in responders at pre-treatment and increased post- treatment.
Kato et al.	(32)	2021	RCC	aPD1	Blood	Responders showed a greater number of blood-tumor overlapping clones at post-treatment.
Snyder et al.	(45)	2017	Urothelial	aPD-L1	Blood	Tumor-overlapping clones expanded more in blood in responders at 3 weeks post-treatment.
Casarrubios et al.	(28)	2021	NSCLC	Neoadjuvant aPD1+chemo	Blood	Responders showed expansion of pre-treatment tumor top 1% clones in blood.
Aoki et al.	(46)	2021	GI	aCD4	Blood	Tumor-overlapping CD8^+^ clones in blood were increased in responders, accompanied by clonal replacement.
Zhang et al.	(27)	2019	NSCLC	Neoadjuvant aPD1	Blood	Tumor T-cell repertoire of responders contained a higher proportion of clones expanded in blood during treatment.

^A^CRC, Colorectal cancer; GI, Gastrointestinal cancer; RCC, Renal cell carcinoma; NSCLC, Non-small cell lung cancer.

^B^Organs analyzed other than tumor.

In a preclinical study, we demonstrated the increased dLN-tumor overlapping repertoire in B16-bearing mice receiving an anti-CD4 depleting antibody treatment (42). Anti-CD4 depleting antibody exhibits a potent anti-tumor effect by transiently removing CD4^+^ immunosuppressive cells, including regulatory T cells, and activating tumor-reactive CD8^+^ T cells (47). While the clonality of CD8^+^ T cells in the tumor was equivalent between the untreated and anti-CD4-treated mice, the total frequency and diversity of the dLN-tumor overlapping repertoire were significantly increased in the anti-CD4-treated mice. In addition, the expansion of adoptively transferred or endogenous melanoma-reactive clones was detected in the dLN-tumor overlapping repertoire (42). These results support that treatment-induced changes in TCR repertoire are enriched in dLN-tumor overlap. Moreover, the dLN-tumor overlapping clones could be classified into three patterns: “Tumor^Major^ clones”, which were dominant (>0.1%) in the tumor, but not in the dLNs; “dLN^Major^ clones”, which were dominant in the dLNs, but not in the tumor; and “Double^Major^ clones”, which were dominant in both the dLNs and the tumor (42). Anti-CD4 treatment increased the dLN^Major^ and Double^Major^ clones, which strengthens the evidence that the primary target of anti-CD4 antibody in the cancer-immunity cycle is priming the tumor-reactive T cells in dLN. The anti-CD4 antibody produced a synergistic anti-tumor effect in combination with PD1 blockade in a preclinical model (47). Considering that the primary target of PD1 blockade is preventing tumor-reactive T cells from exhaustion in the tumor (38), other treatments that increase dLN-tumor overlap may also work synergistically with PD1 blockade by targeting dLN and mobilize more tumor-reactive clones.

### Blood-Tumor Overlapping Repertoire Analysis

A number of studies reported the association between blood-tumor overlapping repertoire and clinical responses (**Table 4**). Chow et al. reported that Tumor^Major^ clones in blood-tumor overlap expanded in the blood within two weeks after initiating stereotactic body radiation therapy (43). In addition, responders for PD-1 blockade therapy showed a higher total frequency of blood-tumor overlapping clones in the blood before treatment (44), a greater number of blood-tumor overlapping clones after treatment (32), and more pronounced expansion of blood-tumor overlapping clones in the blood after treatment (45). Similar observations were reported for neoadjuvant chemoimmunotherapy (28) and clinical trial of a humanized anti-CD4 antibody (46, 48). The association between the blood-tumor overlapping repertoire in the tumor and clinical responses has also been analyzed. Zhang et al. reported that, in a trial of neoadjuvant PD-1 blockade therapy for NSCLC, the tumor T-cell repertoire of responders contained a higher proportion of clones that expanded in the blood after treatment than that of non-responders (27). Moreover, Yost et al. showed that T-cell clones in the tumor were replaced during treatment and that some of the newly emerged clones in the tumor were also present in the peripheral blood before treatment (49).

These observations suggest that PD-1 blockade therapy activates novel tumor-reactive T-cell clones outside the tumor and promotes their infiltration into the tumor (50). This “tumor-extrinsic” response may coincide with the reactivation of exhausted T cells in the tumor, which has been previously considered the central mechanism of PD-1 blockade. In addition, scTCR analysis of tumors and peripheral blood (see below) has shown that genes related to effector function are highly expressed in tumor-overlapping clones in the peripheral blood (51, 52). These results suggested the potential of tumor-overlapping clones in the peripheral blood as a prognostic and early diagnostic marker for PD-1 blockade therapy: patients with more tumor-overlapping clones may benefit from PD-1 blockade, while those with less tumor-overlapping clones may need more aggressive immunotherapy like a combination with anti-CTLA4 antibody. Moreover, whether these overlapping clones indeed recognize tumor cells and contribute to the antitumor effect will require verification in preclinical and clinical studies.

## Overview of Single-Cell TCR Sequencing Methods

In the TCRseq methods described above, the total DNA or RNA in a T-cell population is pooled for library preparation (bulk TCRseq). Therefore, it is impossible to associate a gene expression profile with a particular clone, which poses a major hurdle to understanding the function of T-cell clones in TCR repertoire analysis.

scTCRseq is a potent approach to assigning immunological phenotypes to individual T-cell clones (11). scTCRseq identifies paired TCR alpha and beta sequences of thousands of T cells in parallel using unique DNA barcodes for individual cells (cellular barcodes) (53, 54). Single-cell gene expression profiles can be obtained simultaneously (scRNAseq), and the scRNAseq and scTCRseq results can be integrated using cellular barcodes. By extracting T cells with a particular TCR sequence using the cellular barcode and analyzing the gene expression profile, the phenotypes of individual T-cell clones (including naïve, effector, memory, and exhausted T cells) can be evaluated.

**Figure 3:**
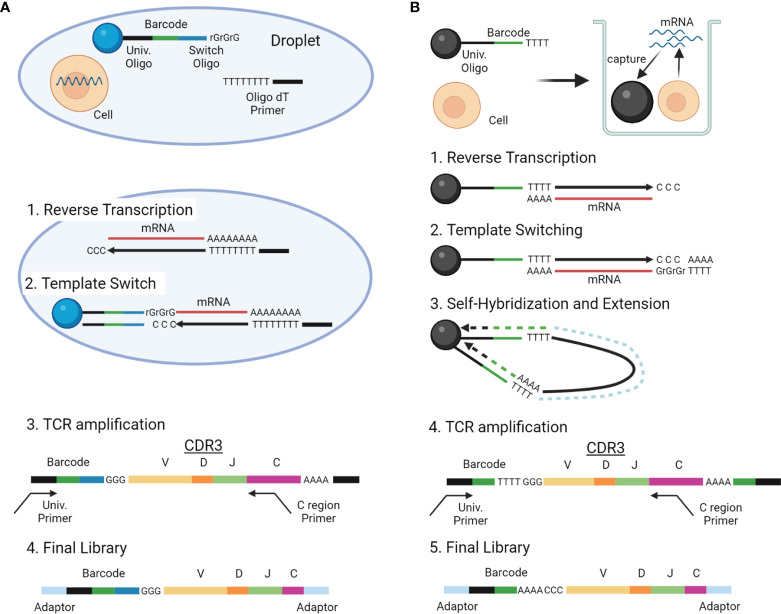
Overview of scTCRseq methods. **(A)** Overview of the scTCRseq method using 5′ RNA amplification. Cells are isolated in oil-in-water droplets. After cell lysis, mRNAs from a single cell are reverse-transcribed using oligo-dT primers. An adapter containing a cellular barcode and a universal oligo is added at the 5′ end of cDNA by template switching. TCR transcripts containing CDR3 and the cellular barcode are specifically amplified using primers designed to target the TCR C region and the universal oligo. The amplified TCRs are sequenced using NGS. **(B)** Overview of the scTCRseq method using self-hybridization. Single cells and beads with cellular barcodes are loaded into micro-wells. After cell lysis, mRNAs from the single cells are captured by the oligo-dT sequences on the beads. After reverse transcription, a synthetic poly-A tail is added to the 3′ end of the cDNA by template switching. Through denaturation and annealing, the synthetic poly-A tail at the 3′ end of the cDNA hybridizes to the oligo-dT sequence on its own bead. The second strand of cDNA is then stretched by DNA polymerase to copy the cellular barcode of the bead. TCR transcripts containing CDR3 and a cellular barcode are specifically amplified using primers designed to target the TCR C region and the universal oligo. The amplified TCRs are sequenced using NGS.

With the advancements in single-cell analysis, more high-throughput scTCRseq methods are being developed, which can be roughly divided into two types: (1) methods reconstructing TCR sequences by extracting TCR reads from standard scRNAseq data and (2) those amplifying TCR genes selectively from the scRNAseq library and sequencing both the TCR gene and scRNAseq libraries. Experimental overview and characteristics of these two categories of scTCRseq methods are summarized in **Figure 3** and **Tables 2**, **5** (17, 18, 55, 56).

**Table 5:** Classifications of TCRseq methods and their characteristics.

Overview	Characteristics	Computational tools and experimental methods	Ref.
Extract TCR reads from scRNAseq data	(+) Additional library preparation is not required and existing scRNAseq data can be used.	TraCeR	(17)
		VDJPuzzle	(18)
Selective amplification of scTCR library	(+) High TCR detection rate (up to 80–90%) can be achieved with a relatively small read count	5′ RNA amplification (10X Chromium^A^)	(55)
		Self-hybridization (BD Rhapsody^A^)	(56)

^A^Single cell analysis platforms in which the indicated experimental method can be used.

## Application of scTCR Analysis to Elucidating T-Cell Responses in the Tumor Microenvironment

One of the applications of scTCRseq in tumor immunology is estimating the developmental relationships among T-cell subsets based on the T-cell clone overlap. scRNAseq has identified several clusters of tumor-infiltrating CD8^+^ T cells (36, 57–61), including a cytotoxic cluster that expresses high levels of perforin and granzyme, an exhausted cluster that is highly tumor-responsive, but expresses high levels of inhibitory receptors, and a tissue-resident memory cluster that expresses integrin alpha E. The exhausted cluster is further classified into a terminally exhausted cluster, which expresses high levels of inhibitory receptors, and a progenitor exhausted cluster, which expresses molecules related to self-renewal capacity, such as TCF1. However, the developmental relationship between these T-cell clusters has not been clarified.

The differentiation trajectory of TILs can be estimated by quantifying the overlapping T-cell clones among the clusters. T cells within a particular TCR clone are derived from a single naïve T cell. Thus, the presence of multiple overlapping clones between two clusters suggests that they are closely related in terms of differentiation trajectory. For example, Li et al. found a considerable overlap of T-cell clones between the late and early exhaustion clusters of CD8^+^ TILs in melanoma, whereas there was limited clonal overlap between the cytotoxic cluster and these exhaustion clusters (57). This finding indicated that the exhaustion and cytotoxic clusters have different differentiation trajectories. In addition, TCR overlap analysis in NSCLC suggested that terminally exhausted CD8^+^ T cells in tumors had two origins: GZMK^+^ circulating precursors infiltrating from the peripheral blood and XCL1^+^ resident precursors showing tissue-resident characteristics (35, 62). Inter-cluster TCR overlap analysis will be a useful method for inferring the differentiation trajectories of T cells *in silico* in addition to trajectory analysis based on gene expression. However, the number of T-cell clones that can be analyzed by scTCRseq is limited. Therefore, a higher throughput method, such as bulk TCRseq of specific T-cell subsets, will be required to validate scTCRseq results in the future.

Another application of scTCRseq is the identification of TCRs that respond to tumor antigens. Tumor-infiltrating T cells include not only tumor-reactive T-cell clones that recognize tumor antigens, but also clones that recognize viral antigens unrelated to tumors (63, 64). Since the antigen specificity of a T-cell clone is determined by the TCR alpha and beta chains, scTCRseq identifying the TCR alpha and beta chain pair is required to reconstitute TCRs of T-cell clones and examine their tumor-reactivity. Oliveira et al. identified TCR alpha- and beta-chain pairs of CD8^+^ TILs by scTCRseq and introduced them into peripheral blood T cells using lentiviral vectors. The antigen specificity of the TCRs was identified by coculturing the TCR-transfected T cells with immortalized lymphoblastoid cell lines pulsed with peptides of tumor antigens or tumor-unrelated viral antigens. In addition, the gene expression profiles of the T-cell clones were determined by scRNAseq. Using this approach, the authors found that the clones recognizing tumor antigens showed an exhaustion phenotype, whereas the bystander clones recognizing viral antigens showed a memory-like phenotype (65). Tumor-reactive TCRs identified by scTCRseq as above can be applied to tailor-made adoptive T-cell therapy. A recent study demonstrated that only a small percentage of TILs can be cultured *ex vivo* (66). Considering this, direct identification of functional T-cell clones using scTCRseq will expand the choice of T-cell clones for T-cell therapy, which will lead to improved therapeutic outcomes.

## Conclusion

The repertoire of tumor-infiltrating T cells, which reflects the antigen-specific T-cell-mediated anti-tumor responses, is an important characteristic feature of the tumor microenvironment. In this review, we introduce two novel approaches for immunological interpretation of the TCR repertoire. Inter-organ clone tracking analysis based on bulk TCRseq can enrich tumor-reactive T-cell clones that are mobilized into the cancer-immunity cycle, providing a more direct index of the antitumor T-cell response than repertoire diversity or clonality. scTCRseq technology enables linking individual T-cell clones with their gene expression profiles, allowing an improved immunological interpretation of the TCR repertoire. Moreover, high-throughput identification of antigen-specific T cells is becoming possible by combining scTCRseq and DNA-barcoded peptide-MHC multimer technology (56, 67, 68). While scTCRseq provides a more precise characterization of individual clones, the number of T-cell clones that can be analyzed per sample is only the “tip of the iceberg” of the TCR repertoire. Thus, combined application of high-throughput bulk repertoire overlap analysis and scTCRseq will improve our understanding of the antitumor responses of diverse T-cell clones. Overall, the approaches for TCR repertoire analysis described in this review will not only provide a more precise characterization of the tumor microenvironment, but also help develop efficient immunotherapeutic strategies to combat cancer.

## Author Contributions

HA and SU wrote the initial draft of manuscript. All authors contributed to the article and approved the submitted version.

## Funding

This work was supported by the Japan Society for the Promotion of Science under Grant Number 20281832 and 17929397, and by the Japan Agency for Medical Research and Development (AMED) under Grant Number JP 21gm6210025 and JP21fk0210049.

## Conflict of Interest

HA reports stock for ImmunoGeneTeqs, Inc. SU reports advisory role for ImmunoGeneTeqs, Inc; stock for ImmunoGeneTeqs, Inc, IDAC Theranostics, Inc. SS reports advisory role for ImmunoGeneTeqs, Inc; stock for ImmunoGeneTeqs, Inc, KM reports consulting or advisory role for Kyowa-Hakko Kirin, ImmunoGeneTeqs, Inc; research funding from Kyowa-Hakko Kirin, and Ono; stock for ImmunoGeneTeqs, Inc, IDAC Theranostics, Inc.

## Publisher’s Note

All claims expressed in this article are solely those of the authors and do not necessarily represent those of their affiliated organizations, or those of the publisher, the editors and the reviewers. Any product that may be evaluated in this article, or claim that may be made by its manufacturer, is not guaranteed or endorsed by the publisher.
